# Regulation of Antitumor Immune Responses by the IL-12 Family Cytokines, IL-12, IL-23, and IL-27

**DOI:** 10.1155/2010/832454

**Published:** 2010-09-14

**Authors:** Mingli Xu, Izuru Mizoguchi, Noriko Morishima, Yukino Chiba, Junichiro Mizuguchi, Takayuki Yoshimoto

**Affiliations:** ^1^Intractable Disease Research Center, Institute of Medical Science, Tokyo Medical University, 6-1-1 Shinjuku, Shinjuku-ku, Tokyo 160-8402, Japan; ^2^Department of Immunology, Tokyo Medical University, 6-1-1 Shinjuku, Shinjuku-ku, Tokyo 160-8402, Japan

## Abstract

The interleukin (IL)-12 family, which is composed of heterodimeric cytokines including IL-12, IL-23, and IL-27, is produced by antigen-presenting cells such as macrophages and dendritic cells and plays critical roles in the regulation of helper T (Th) cell differentiation. IL-12 induces IFN-*γ* production by NK and T cells and differentiation to Th1 cells. IL-23 induces IL-17 production by memory T cells and expands and maintains inflammatory Th17 cells. IL-27 induces the early Th1 differentiation and generation of IL-10-producing regulatory T cells. In addition, these cytokines induce distinct immune responses to tumors. IL-12 activates signal transducers and activator of transcription (STAT)4 and enhances antitumor cellular immunity through interferon (IFN)-*γ* production. IL-27 activates STAT1, as does IFN-*γ* and STAT3 as well, and enhances antitumor immunity by augmenting cellular and humoral immunities. In contrast, although exogenously overexpressed IL-23 enhances antitumor immunity via memory T cells, endogenous IL-23 promotes protumor immunity through STAT3 activation by inducing inflammatory responses including IL-17 production.

## 1. Introduction

### 1.1. Cytokine-Based Cancer Immunotherapy

Tumor cells are characterized by low expression of major histocompatibility complex (MHC) class and costimulatory molecules such as CD80 and CD86. In mice bearing tumors and cancer patients, the production of immune suppressive cytokines such as interleukin (IL)-10 and transforming growth factor (TGF)-*β* is accelerated, and immune regulatory cells such as regulatory T (Treg) cells and IL-10-producing type I Treg (Tr1) cells are highly infiltrated in tumor microenvironment. Thus, tumor cells can escape from the immune surveillance system. As one of the strategies to enhance the antitumor immune responses, it is possible to activate tumor-specific antitumor immune responses by systemic injection of cytokine or introduction of cytokine gene into tumors through activating natural killer (NK) cells and tumor-specific CD4^+^ T cells and cytotoxic T lymphocytes (CTL). In order to localize the effect of cytokine within tumor microenvironment, tumor cells are transfected with cytokine gene and then injected into host. These tumors begin to grow but then regress and are finally rejected. In some cases, intense inflammatory infiltrates accumulate around the cytokine-secreting tumors, and the nature of the infiltrate varies with the cytokine. Different cytokines may stimulate antitumor immune responses by different mechanisms. Importantly, the injection of cytokine-secreting tumors induces T-cell-mediated immunity specific to the parental tumor cells.

### 1.2. Cancer Immunotherapy Using Granulocyte Macrophage Colony-Stimulating Factor (GM-CSF) and IL-2

For instance, GM-CSF, which acts on bone marrow cells and differentiates and matures them to neutrophils, monocytes, and dendritic cells (DCs), is used to generate the cancer immunotherapy called GAVX [1, 2]. The tumor cells, transfected with GM-CSF gene, have been demonstrated to induce potent, long-lasting, tolerance-breaking, and tumoricidal immune responses in a variety of tumor models including poorly immunogenic tumors. In clinical trials using the GAVX, induction of systemic antitumor immune responses and clinical activity was observed in renal cell carcinoma, melanoma, pancreatic cancer, and prostate cancer. Moreover, IL-2 is a growth factor for T lymphocytes and NK cells. In the beginning of 1980s, lymphokine-activated killer (LAK) therapy was developed by Rosenberg and his colleagues [3], and high-dose bolus schedules of IL-2 showed promising effects in metastatic renal cell carcinoma with LAK cells or tumor infiltrating lymphocytes [4]. Subsequently, the administration of high-dose bolus IL-2 has consistently induced durable responses in a small percentage of patients with advanced renal cell carcinoma [5].

### 1.3. Cancer Immunotherapy Using Novel Cytokines

**Figure 1:**
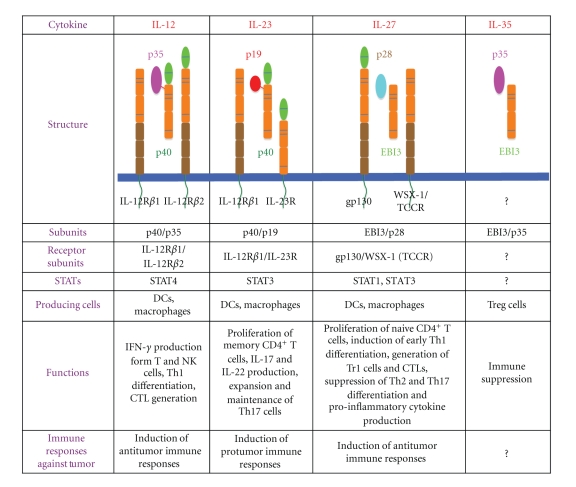
Molecular characterization of the IL-12 family cytokines, IL-12, IL-23, IL-27, and IL-35. IL-12 induces IFN-*γ* production by NK and T cells and differentiation to Th1 cells. IL-23 induces IL-17 production by memory T cells and expands and maintains inflammatory Th17 cells. IL-27 induces the early Th1 differentiation and generation of IL-10-producing Tr1 cells. In addition, these cytokines induce distinct immune responses to tumors. IL-12 activates STAT4 and enhances antitumor cellular immunity through IFN-*γ* production. IL-27 activates STAT1, as does IFN-*γ* and STAT3 as well, and enhances antitumor immunity by augmenting cellular and humoral immunities. In contrast, although exogenously overexpressed IL-23 enhances antitumor immunity via memory T cells, endogenous IL-23 promotes protumor immunity through STAT3 activation by inducing inflammatory responses including IL-17 production.

Cancer immunotherapy has thus the potential of being the most tumor-specific treatment that can be devised. More recently, several exciting cytokines have been characterized that have considerable promise for future cancer immunotherapy. The identification of new cytokines will open up a novel avenue to develop the cancer immunotherapy. So far, cytokines up to IL-35 have been identified. Among them, the IL-12 family cytokines have quite unique properties that they are heterodimeric cytokines and produced by antigen-presenting cells such as macrophages and DCs, and play critical roles in the regulation of helper T (Th) differentiation (Figure 1) [6]. Although IL-12 is one of the most powerful antitumor cytokine [7, 8], accumulating evidence revealed that the individual members of the IL-12 family play distinct roles in the regulation of antitumor immune responses.

### 1.4. Regulation of Antitumor Immune Responses by Inflammation

While immune surveillance thus induces antitumor immune responses, chronic inflammation has long been considered to be associated with increased incidence of malignancy [9]. Recently, substantial numbers of evidence indicate that immune system also promotes the tumorigenesis, invasion, propagation, and metastasis of tumors [10, 11]. Moreover, infiltration of effector cells such as CTLs in tumors generally results in good prognosis, while infiltration of macrophages often leads to bad prognosis. More recently, the molecular mechanism whereby the inflammation regulates the antitumor immune responses has been elucidated [12]. In many tumors, signal transducers and activator of transcription (STAT)3 are activated [13], and thereby IL-12 production is inhibited, and antitumor immune surveillance is suppressed. On the contrary, the production of IL-23, which plays critical roles in expansion and maintenance of Th17 cells producing inflammatory IL-17, is augmented through STAT3 activation, resulting in inflammation, which is advantageous in the promotion of tumorigenesis.

In the present paper, we summarize the recent advance on the regulation of antitumor immune responses by the IL-12 family cytokines, IL-12, IL-23, and IL-27.

## 2. IL-12

### 2.1. Molecular Characterization of IL-12

IL-12 was independently identified as natural killer-stimulating factor (NKSF) and cytotoxic lymphocyte maturation factor (CLMF) by Trinchieri's group in 1989 [14] and Gately's group in 1990 [15], respectively. Its cDNA was then cloned in 1991 [16] and named IL-12. IL-12, which consists of p40 and p35 subunits, induces proliferation of NK and T cells and production of cytokines, especially IFN-*γ*, and also enhances the generation and activity of CTLs, through activation of STAT4 [17, 18]. IL-12 is an essential cytokine for the differentiation to Th1 cells, which is required for the generation of type 1 cell-mediated immunity to cancer and infection. IFN-*γ*, produced by IL-12, then upregulates the expression of MHC class I and II molecules, adhesion molecules such as intracellular adhesion molecules (ICAM)-1 and transcription factors such as T-box expressed in T cells (T-bet). IL-12 also induces the production of antiangiogenic chemokines such as IFN-*γ*-inducible protein (IP-10, CXCL10) and monokine-induced by IFN-*γ* (MIG, CXCL9) in endothelial cells [8]. Of note, the endogenous production of IFN-*γ* is required for the antitumor effect of IL-12 in most, if not all, cases [19–21].

### 2.2. Potent Antitumor Activity of IL-12

The antitumor and antimetastatic activities of IL-12 have been extensively examined in a variety of murine tumor models including melanomas, mammary carcinomas, colon carcinoma, renal carcinoma, and sarcomas [7, 8]. For instance, administration of IL-12 into tumor-bearing mice can delay, reduce, and, in some cases, completely inhibit tumor development with significant therapeutic efficacy in many solid tumors as well as hematological leukemias and lymphomas. In clinical trials, however, the therapeutic effect has been limited by low efficacy and systemic toxicities such as splenomegaly, leucopenia, and gastrointestinal toxicity [21–23]. Although the reasons for the limited clinical efficacy of IL-12 in cancer patients remain incompletely understood, several immunosuppressive mechanisms including a Th1 to Th2 shift due to increased IL-10 production and diminished IFN-*γ* production after repetitive treatments with IL-12 could be involved [24, 25]. In addition, immune suppressive microenvironment characterized by infiltration of Treg cells and Tr1 cells in advanced tumors [26] could contribute to the limited efficacy. To improve the therapeutic efficacy with IL-12 but simultaneously minimize the toxicity, several strategies have been created including targeting of IL-12 to only tumor, and coadministration with Treg cell-depleting antibodies such as anti-CD25 antibody, antibodies against immune suppressive signals such as cytotoxic T lymphocyte antigen (CTLA)-4 and IL-10, other cytokines, and anticancer drugs. Thus, IL-12 is one of the most potential cytokine for cancer immunotherapy.

## 3. IL-27

### 3.1. Molecular Characterization of IL-27

IL-27, which has structurally and functionally similarities to IL-12, was identified by Kastelein's group in 2002 [27]. IL-27, which consists of EBI3 subunit and the IL-12p35 subunit, activates both STAT1 and STAT3 through distinct IL-27 receptor (R) subunits, WSX-1 and gp130, respectively, and enhances proliferation of naive CD4^+^T cells [27–30]. IL-27 promotes the early Th1 differentiation through upregulation of ICAM-1 [30] and T-bet [28], but it suppresses the differentiation to Th2 [31, 32] and Th17 [33, 34] and production of pro-inflammatory cytokines [35, 36]. One possible mechanism for the suppressive function is considered to be mediated through IL-10 production [37–39]. In addition, IL-27 plays a dominant function together with TGF-*β* in generating IL-10-producing anti-inflammatory Tr1 cells [40].

### 3.2. Antitumor Activity of IL-27 Mediated by CD8^+^ T Cells

**Figure 2:**
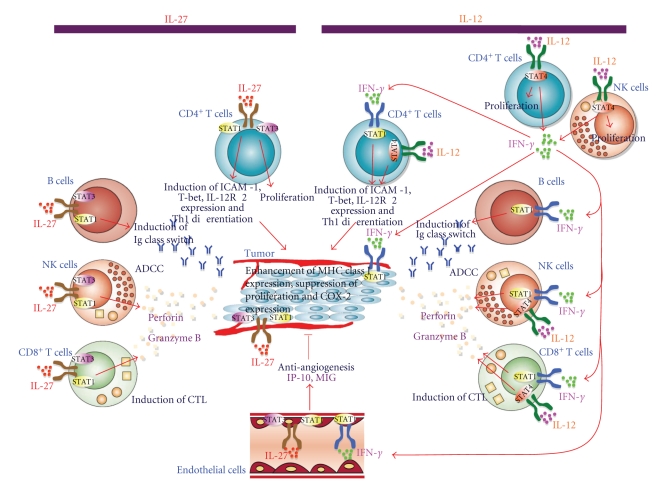
Differences in the molecular mechanisms to induce antitumor effects between IL-12 and IL-27. IL-27 utilizes several mechanisms to induce antitumor effects depending on characteristics of individual tumors. Against highly immunogenic tumors expressing MHC class I, IL-27 exerts antitumor effects by mainly CD8^+^ T cells. However, against poorly immunogenic tumors lacking expression of IL-27R, IL-27 has to use other mechanisms including NK cells and antiangiogenesis to exert its antitumor effects. In contrast, IL-12 has a strong antitumor effect through high production of IFN-*γ* by NK and T cells. This high IFN-*γ* production also causes systemic toxicities, which leads to limitation of the IL-12 therapy in clinical trials.

Since Hisada et al. first evaluated the antitumor efficacy of IL-27 in 2004 [41], accumulating evidence has revealed that IL-27 has a potent antitumor activity, which is mediated by a variety of mechanisms including CD8^+^T cells [41–45], NK cells [46–48], antibody-dependent cell-mediated cytotoxicity (ADCC) [49], antiangiogenesis [50], direct suppression of tumor growth [51], and inhibition of cychroxygenase-2 (COX-2) expression [52], depending on the characteristics of individual tumors (Figure 2). Because each subunit of IL-27 is not chemically bound like IL-12 and IL-23, a single chain expression vector was constructed using flexible linker such as (Gly_4_Ser)_3_ and resultant recombinant fusion protein was prepared [27, 41]. For instance, highly immunogenic murine colon carcinoma Colon 26 cells transfected with the IL-27 expression vector showed greatly reduced tumor growth, which is mainly mediated by CD8^+^ T cells, IFN-*γ* and T-bet [41]. In addition to CD4^+^ T cells, IL-27 directly acts on CD8^+^ T cells to induce the expression of transcriptional factors, T-bet and Eomesodermin (EOMES) and thereby augments the generation of CTL through enhancing the expression of effector molecules such as granzyme B and perforin [53]. In addition, IL-27 has an adjuvant activity for induction of epitope-specific CTL in a prime-boost immunization [54]. The role of endogenous IL-27 in the generation of CTL and antitumor immunity was also examined using mice deficient in WSX-1, one of the IL-27R subunits [55]. Endogenous IL-27 was revealed to promote tumor-specific CTL generation in CD8^+^ T cells, while suppressing APC function in DCs, during generation of tumor immunity. Thus, IL-27 plays an important role in the induction of CTL [56].

### 3.3. Antitumor Activity of IL-27 Mediated by NK Cells, Angiogenesis, and Its Direct Effects on Tumors

The antitumor effects of IL-27 against poorly immunogenic tumors such as B16F10 melanoma are mediated by various mechanisms through NK cells [46–48], angiogenesis [50], and its direct effects on tumors [51, 52]. IL-27 not only activates NK cells but also induces tumor-specific immunoglobulin that cooperatively elicit ADCC activity [49]. IL-27 also possesses potent antiangiogenic activity on melanomas as does IFN-*γ*, which contributes to its antitumor and antimetastatic activities [50]. B16F10 cells transfected with IL-27 gene exert antitumor activity against subcutaneous tumor and also experimental pulmonary metastasis even in IFN-*γ*-deficient mice. Moreover, in immunodeficient NOD-SCID mice lacking a functional immune system including T, B, and NK cells, the antitumor activities are decreased, but they are still fairly well retained by exogenous expression of IL-27, suggesting that different mechanisms other than the immune response are also involved. B16F10 cells transfected with IL-27 gene not only markedly suppress tumor-induced neovascularization in lung metastases but also clearly inhibit angiogenesis. Consistent with these results, IL-27 was revealed to directly act on human umbilical vein endothelial cells and induce production of the antiangiogenic chemokines such as IP-10 and MIG. IL-27 also possesses potent direct antiproliferative activity on melanomas as does IFN-*γ* [51]. Although parental B16F10 cells express only gp130 but not WSX-1 IL-27 induces activation of STAT1 and STAT3 and upregulation of MHC class I in B16F10 transfectants expressing wild-type WSX-1, and inhibits the growth of these tumors as well. Moreover, IL-27 induces the expression of both IFN-regulatory factor (IRF)-1 and -8 whereas IRF-1 but not IRF-8 is partially involved in these effects. Similar direct antiproliferative effect is observed in several human melanomas.

### 3.4. Differences in the Molecular Mechanisms to Induce Antitumor Effects between IL-12 and IL-27

As mentioned above, IL-27 utilizes several mechanisms to induce antitumor effects depending on characteristics of individual tumors. Against highly immunogenic tumors expressing MHC class I, IL-27 exerts antitumor effects by mainly CD8^+^ T cells. However, against poorly immunogenic tumors lacking expression of IL-27R, IL-27 cannot induce antitumor effect by CD8^+^ T cells. This is because IL-27 itself has only weak ability to induce IFN-*γ* production and therefore fails to upregulate MHC class I expression on these tumors. Thus, IL-27 has to use other mechanisms including NK cells and antiangiogenesis to exert its antitumor effects. In contrast, IL-12 has a strong antitumor effect through high production of IFN-*γ* by NK and T cells [7, 8]. This high IFN-*γ* production also causes systemic toxicities, which leads to limitation of the IL-12 therapy in clinical trials [21–23]. In this regards, since IL-27 has much less toxicities compared with IL-12 [41, 46], probably due to much lower ability of IL-27 to produce IFN-*γ* by NK cells, IL-27 may be an attractive candidate as an antitumor agent applicable to cancer immunotherapy.

## 4. IL-23

### 4.1. Molecular Characterization of IL-23

IL-23 was identified by Kastelein's group in 2000 and is composed of the IL-23-specific p19 subunit and the IL-12p40 subunit [57]. IL-23 activates STAT3 and preferentially acts on memory CD4^+^T cells and induces their proliferation and production of cytokines such as IL-17 and IL-22 [58, 59]. Although p19-deficient mice are highly resistant to the development of experimental allergic encephalomyelitis (EAE) [60], the physiological roles of IL-23 is considered to only expand and maintain the inflammatory Th17 cells [61], and TGF-*β* and IL-6 are necessary to induce the Th17 differentiation [62, 63].

### 4.2. Antitumor Effect of Exogenously Overexpressed IL-23

Murine colon carcinoma CT26 and B16F10 tumor cells transfected with IL-23 gene show potent antitumor and antimetastatic effects similar to those of IL-12 [46, 64, 65]. However, the antitumor mechanisms induced by IL-12 and IL-23 are quite different. IL-23-mediated tumor suppression is only evident at later time points after tumor inoculation, while IL-12-induced tumor suppression is obvious in an earlier time points [46, 64]. Since the protective effects of IL-23 are completely abrogated in mice depleted of CD8^+^ T cells, CD8^+^ T cells play an important role in the IL-23-mediated antitumor activity. Moreover, when high-dose IL-23 is systemically administrated into the pretibial muscles using in vivo electroporation of IL-23 plasmid, significant suppression of the growth of preexisting MCA205 fibrosarcoma and prolongation of the survival without any significant toxicity are observed [66]. For these potent antitumor effects of IL-23 treatment, fully promoted Th1-type response in the presence of endogenously expressed IL-12 is necessary. Thus, exogenously overexpressed IL-23 in tumor cells induces a potent antitumor effect through memory T cells.

### 4.3. Protumor Effect of Endogenous IL-23

**Figure 3:**
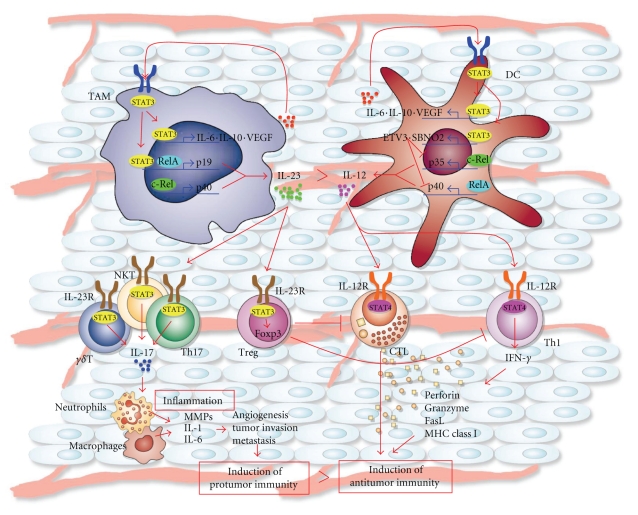
Differences in the immune responses to tumor induced by IL-12 and IL-23 and their molecular mechanisms in tumor microenvironments. STAT3 activation induces IL-23 expression, which is mainly observed in TAMs, via direct transcriptional activation of the IL-23p19 gene together with NF-*κ*B/RelA(p65). On the other hand, STAT3 activation inhibits NF-*κ*B/c-Rel-dependent IL-12p35 gene expression in tumor-associated DCs, possibly through the STAT3-dependent induction of the ETS transcriptional suppressor ETV3 and the helicase family corepressor SBNO2. Thus, STAT3 promotes IL-23-mediated protumor immune responses while inhibiting IL-12-dependent antitumor immunity.

Contrary to the exogenous IL-23, endogenous expression of IL-23 has been reported to promote tumor incidence and growth, and there are strikingly differences in the regulation of immune surveillance to tumors between IL-12- and IL-23-deficient mice (Figure 3) [10, 11]. IL-12 deficiency increases not only the incidence of tumors but also allows for rapid tumor growth in mice. By contrast, deficiency in IL-23 or the IL-23R not only dramatically reduces tumor incidence but also reduces tumor growth of established tumors [10]. In the local tumor microenvironment, IL-23 not only induces the hallmarks of chronic inflammation such as matrix metalloproteases (MMPs), angiogenesis, and macrophage infiltration, but also reduces antitumor immune surveillance by locally suppressing the presence of CD8^+^ T cells [10]. By contrast, the absence of IL-12 leads to exacerbation of the myeloid-driven inflammation with a coincident lack of CD8^+^ T cells. Of note, IL-23p19 and IL-12p40, but not IL-12p35, are overexpressed in the majority of human cancers. Taken together, endogenous IL-23 expression promotes tumor incidence and growth, while application of IL-23 at the excessive amount induces antitumor immune responses with the characteristics immune responses mediated by the function of CD4^+^ and CD8^+^ T cells.

### 4.4. Promotion of Tumorigenesis by STAT3 Activation

The transcription factor STAT3 is constitutively activated in diverse cancers [13]. Its activation favors proliferation of tumor cells by exerting an antiapoptotic effect, in part, mediated by transcriptional downregulation of p53 and by inducing factors that drive angiogenesis and metastasis including vascular endothelial growth factor (VEGF) and MMPs [67]. Several immunosuppressive factors including IL-10 are also produced. Although STAT3 activation induces recruitment of hematopoietic cells, STAT3 activation in tumor-associated macrophages (TAMs) and DCs has a profound anti-inflammatory effect by preventing their maturation and blocking their ability to produce many proinflammatory cytokines such as IL-12 (Figure 3) [12, 68]. Indeed, progressing tumors in humans and experimental animals are characterized by the presence of infiltrating immature and anergic TAMs and DCs and a limited infiltration of T lymphocytes that often have the characteristic of Treg cells. Treg cells play a role in inactivation of antigen-presenting cells and suppression of proliferation and antitumor activity of effector T cells, including IFN-*γ*-producing Th1 cells and CTLs with antitumor activity, by the production of IL-10 and TGF-*β* or direct cellular contacts [69].

### 4.5. Promotion of IL-23-Mediated Protumor Immune Responses by STAT3 while Inhibiting IL-12-Dependent Antitumor Immune Responses

Interactions between tumor and immune cells either enhance or inhibit cancer progression. STAT3 signaling within the tumor microenvironment was recently elucidated to induce a protumor cytokine, IL-23, while inhibiting a central antitumor cytokine, IL-12, thereby shifting the balance of tumor immunity toward tumorigenesis (Figure 3) [12, 68]. STAT3 activation induces IL-23 expression, which is mainly observed in TAMs, via direct transcriptional activation of the IL-23p19 gene together with NF-*κ*B/RelA(p65). On the other hand, STAT3 activation inhibits NF-*κ*B/c-Rel-dependent IL-12p35 gene expression in tumor-associated DCs, possibly through the STAT3-dependent induction of the ETS transcriptional suppressor, ETS variant gene 3 (ETV3), and the helicase family corepressor, strawberry notch homolog 2 (SBNO2). These transcription factors were previously demonstrated to mediate the inhibition of gene transcription by IL-10-induced STAT3 activation [70]. Interestingly, unlike spleen Treg cells, tumor-associated Treg cells express IL-23R and activates STAT3 in response to IL-23, leading to upregulation of the Treg-specific transcription factor Foxp3 and the immunosuppressive cytokine IL-10. This is surprising because in other settings, STAT3 activation by IL-6 or IL-21 in TGF-*β*-exposed T cells induces downregulation of Foxp3 and upregulation of the transcription factor ROR*γ*t leading to induction of IL-17-producing Th17 cells [71]. These results demonstrate that STAT3 promotes IL-23-mediated protumor immune responses while inhibiting IL-12-dependent antitumor immunity.

## 5. Conclusion

Recently, another novel cytokine, IL-35, which belongs to the IL-12 family, was identified by Vignali's group in 2007 [72]. IL-35 consists of the IL-27 EBI3 subunit and the IL-12p35 subunit, produced by Treg cells, but not effector T cells, after interaction between Treg and effector T cells, and it plays a role in immune suppression. However, the role of IL-35 in the regulation of immune responses in various diseases including cancer and infection remains largely unknown [73]. Collectively, IL-12, IL-23, IL-27, and probably IL-35, which belong to the same IL-12 family, play critical roles in the regulation of antitumor and/or protumor immune responses in respective situation.
